# Sex-specific aspects in patients with oropharyngeal squamous cell carcinoma: a bicentric cohort study

**DOI:** 10.1186/s12885-023-11526-6

**Published:** 2023-11-02

**Authors:** Charlotte Klasen, Nora Wuerdemann, Pauline Rothbart, Johanna Prinz, Hans Nicholaus Casper Eckel, Malte Suchan, Christopher Kopp, Jannik Johannsen, Maria Ziogas, Arthur Charpentier, Christian Ulrich Huebbers, Shachi Jenny Sharma, Christine Langer, Christoph Arens, Steffen Wagner, Alexander Quaas, Jens Peter Klußmann

**Affiliations:** 1 Department of Otorhinolaryngology, Head and Neck Surgery, Medical Faculty, University of Cologne, Kerpener Strasse 62, 50937 Cologne, Germany; 2 Center for Molecular Medicine Cologne (CMMC), Faculty of Medicine and University Hospital Cologne University of Cologne, Robert-Koch-Str. 21, 50931 Cologne, Germany; 3 Department I of Internal Medicine, Center for Integrated Oncology Aachen Bonn Cologne Duesseldorf, Faculty of Medicine and University Hospital Cologne, University of Cologne, Kerpener Strasse 62, 50931 Cologne, Germany; 4 Jean-Uhrmacher-Institute for Otorhinolaryngological Research, University of Cologne, Geibelstrasse 29-31, 50931 Cologne, Germany; 5 Department of Otorhinolaryngology, Head and Neck Surgery, Medical Faculty, University of Giessen, Klinikstrasse 33, Giessen, Germany; 6 Institute of Pathology, Medical Faculty, University of Cologne, Kerpener Strasse 62, 50937 Cologne, Germany

**Keywords:** Human papillomavirus (HPV), Oropharyngeal squamous cell carcinoma (OPSCC), Gender, Epidemiology, Survival, Prognosis

## Abstract

**Background:**

Oropharyngeal squamous cell carcinoma (OPSCC) is the only subgroup of head neck cancer that presents with an increased incidence. Gender-specific studies in other cancer entities have revealed differences in treatment response and prognosis. However, only limited data in OPSCC according to gender and human papillomavirus (HPV) status exist. Therefore, we aimed to investigate sex-specific differences in OPSCC and how these may be distributed in relation to HPV and other risk factors.

**Methods:**

This retrospective, bicentric study included 1629 patients with OPSCC diagnosed between 1992 and 2020. We formed subgroups based on TNM status, American Joint Cancer Committee 8^th^ edition (AJCC8), HPV status, treatment modality (surgery (± radio(chemo)therapy (RCT) vs. definitive RCT) and patient-related risk factors and investigated gender differences and their impact on patients survival via descriptive-,uni- and multivariate analysis.

**Results:**

With the exception of alcohol abuse, no significant differences were found in risk factors between men and women. Females presented with better OS than males in the subgroup T1-2, N + , independent of risk factors (*p* = 0.008). Males demonstrated significant stratification through all AJCC8 stages (all *p* < 0.050). In contrast, women were lacking significance between stage II and III (*p* = 0.992). With regard to therapy (surgery (± R(C)T) – vs. definitive RCT) women treated with surgery had better OS than men in the whole cohort (*p* = 0.008). Similar results were detected in the HPV-negative OPSCC sub-cohort (*p* = 0.042) and in high-risk groups (AJCC8 stage III and IV with M0, *p* = 0.003).

**Conclusion:**

Sex-specific differences in OPSCC represent a health disparity, particularly according to staging and treatment, which need to be addressed in future studies.

**Supplementary Information:**

The online version contains supplementary material available at 10.1186/s12885-023-11526-6.

## Background

Head and neck squamous cell carcinoma (HNSCC) constitute the seventh leading type of malignancy worldwide with approximately 878.000 new cases and over 444.000 deaths annually [1]. Oropharyngeal squamous cell carcinoma (OPSCC) is the only subgroup of head neck cancer that presents with an increased incidence [2–4]. Major risk factors are tobacco and alcohol consumption [5, 6]. Over the last two decades, a significant increase in incidence of Human papillomavirus (HPV) related- OPSCC has been observed, in particular in high-income countries [3, 4, 7, 8]. The majority of these OPSCC are associated with high-risk HPV type 16 [9, 10]. Notably, patients with HPV-related OPSCC have a significantly improved prognosis compared to HPV-negative OPSCC, regardless of treatment modality or tumor stage [11–13].

In general, men are diagnosed with OPSCC much more frequently compared to women (approximately 70% vs. 30%) [14]. Furthermore, in a multicenter study including patients from 29 different countries, Castellsagué and colleagues [15] demonstrated that there is a spatial heterogeneity regarding HPV prevalence and distribution of HPV-related OPSCC according to gender. In the US population the rise in incidence is predominantly attributable to male patients [7, 16], whereas in Germany a higher increase in females was reported [4].

Despite these gender differences in incidence, the patient’s sex is usually not considered in diagnostic procedures, classification or clinical decisions. For many years, gender medicine was a neglected topic in oncology. Meanwhile, further differences in diagnosis, tumor aggressiveness and outcome are known in many cancer entities as papillary thyroid cancer and gastric cancer [17, 18]. Various reasons such as differences in habits (smoking, drinking and sexual behavior), differences according to the immune system, molecular differences or hormonal influences are discussed [2, 17–20].

Regarding head and neck cancer one explanation for the above described gender disparities is the difference in habits (more smoking and drinking in men) [6, 21]. Regarding HPV related OPSSC one cause might be the cervicovaginal microbiota. It has been reported that females display a higher viral load of the genital mucosa compared to men, despite similar genital HPV prevalence [22, 23]. Consistent with these findings, other studies have reported that HPV may be transmitted more often from female to male, than vice versa [24, 25] and that there are higher rates of HPV transmission via vaginal–oral rather than penile–oral sex [26]. A higher prevalence of oncogenic HPV in the oral cavity of men compared to women was also detected (10.1% vs. 3.6%) [2, 27], which could in part explain the higher prevalence of HPV-related OPSCC in men [2, 28]. Another reason for a higher HPV prevalence of men might be a higher number of sexual partners [2, 3, 29]. This is in line with the finding of a higher diffusion among (non vaccinated) men having sex with men [30–33].

Independent of HPV infection, another reason for gender disparities in HNSCC might be caused by differences in sexual hormones. Hormones play an important, mostly protective role in different types of cancer in women like hepatocellular carcinoma [34]. A case–control study by Hashim et al. demonstrated that the risk of developing HNSCC was inversely correlated with endogenous and exogenous estrogen exposures [35]. Regarding endogenous hormone exposure, the author specified that women giving birth to a child before 35 years of age had a lower risk of HNSCC than older women or women that have never been pregnant. Furthermore, female hormone pathways can be affected by smoking and alcohol drinking [35, 36]. Smoking is known to increase estrogen catabolism [31], which may be one reason for a different effect of smoking on the risk of developing HNSCC in women than in men.

Here, we performed a bicentric, retrospective analysis of OPSCC patients focused on gender-related overall survival and therapy, with the aim to identify sex-specific differences with potential impact on staging and treatment in the future.

## Materials and methods

### Patient cohort

The study protocol was approved by the Ethics committee of Giessen and Cologne (study number 144/22 Giessen, 19–1288 Cologne). Informed consent was obtained from all the participants and/or their legal guardians. All study procedures were conducted according to the guidelines of the Declaration of Helsinki.

Patients diagnosed with OPSCC (C09, C10, International Classification of Diseases for Oncology (ICD-O)) and treated at the University Hospital Giessen and Cologne between 1992 and 2020 were included in this study. The following data were assessed: Age at initial diagnosis, TNM, ECOG, HPV-status, alcohol and nicotine consumption, treatment and OS. Due to the wide timespan, data was not available in all cases. Therefore, statistical analysis was performed based on available data in each category. The numbers of included cases were specified in each category. The extent of the disease was defined by TNM 7^th^ or 8^th^ edition, according to validity at the time of diagnosis. T4a- and T4b-status in patients with HPV-negative OPSCC were combined into a T4-status for better comparability to patients with HPV-related OPSCC. In terms of N-status, stages N2 a-c and N3 a-b in HPV-negative OPSCC were merged into stages N2 and N3. Based on TNM, the classification according to the 8^th^ edition of the American Cancer Staging Classification, (AJCC8 I-IV) was determined. This was feasible for 1149 patients, whereas information on AJCC8 status was lacking for the remaining cases.

Patients were considered non-smokers if nicotine consumption was suspended 16 years before the initial diagnosis of the OPSCC. Alcohol consumption was marked positive when patients reported a regular alcohol consumption.

**Fig. 1:**
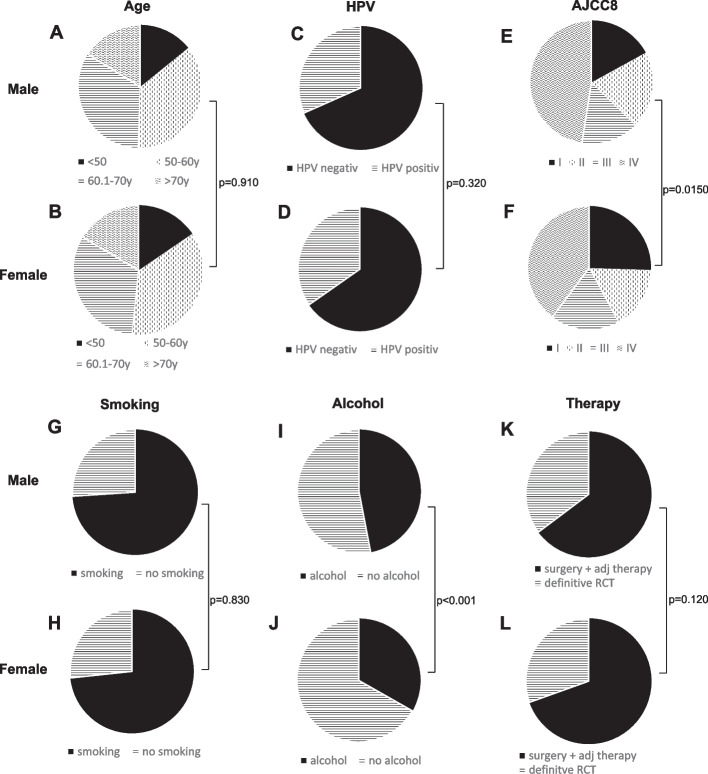
Distribution of patient- and tumor-characteristics, risk factors and therapy modality in OPSCC stratified by sex. Age (**A**, **B**); Human Papillomavirus (HPV) status (HPV-positive defined as HPV + p16 + ; HPV-negative defined as either p16-/HPV-, p16-/HPV + or p16 + /HPV-) (**C**, **D**); tumor stage (American Joint Committee on Cancer 8th edition (AJCC8) (**E**, **F**); smoking (**G**, **H**) and alcohol status (**I**, **J**) and treatment modality (**K**, **L**) in male (**A**, **C**, **E**, **G**, **I**, **K**) and female patients (**B**, **D**, **F**, **H**, **J**, **L**). Gender specific differences were analyzed with Chi-Quadrat test. RCT: radiochemotherapy

Treatment options were divided into either surgery with risk-adapted adjuvant radio(chemo)therapy (R(C)T) versus definitive RCT. Treatment was defined to be the first course of cancer specific therapy. For the analysis of therapeutic differences, only patients with M0 were included in the statistical analysis. Clinicopathological features of the cohort are displayed in Table 1 and Fig. 1.

### HPV status of OPSCC

The formalin-fixed, paraffin-embedded samples containing sufficient tumor tissue either acquired by diagnostic biopsies (in the case of non-surgical treatment) or surgery were all analyzed for the presence of HPV DNA, HPV genotypes and expression of p16^INK4a^ (p16) by immunostaining as described previously [9]. HPV-positivity was defined as a combination of an expression of p16 in more than 70% of tumor cells and high-risk HPV-DNA detection as described previously [9]. HPV-negativity was defined as either p16 negative and HPV-DNA negative (p16-/HPV-), p16 negative and HPV-DNA positive (p16-/HPV +) or p16 positive but HPV-DNA negative (p16 + HPV-). Only when analyzing the OS according to AJCC8^th^ edition (Fig. 3) patients were subdivided solely by their p16 status (independent of HPV-DNA), as defined in the AJCC8 classification criteria.

### Statistical analysis

Statistical analyses were performed using SPSS statistical software (IBM SPSS 28.0, Armonk, NY, USA). Survival curves were plotted according to the Kaplan–Meier method and analyzed using the log-rank test. Patients for whom the time of death or survival could not be determined were censored at the last known contact. Censoring was indicated in the graphs by vertical bars. To assess significant differences in OS, Cox proportional-hazards models were used to estimate hazard ratios (HR) with a confidence interval (CI) of 95%. This was performed as univariate and multivariate analysis. All tests were two-sided. For all tests, p-values < 0.05 were considered statistically significant. Graphs were created using GraphPadPrism (GraphPadPrism 8.3.0, San Diego, CA, USA).

## Results

### Patients characteristics

This retrospective, bi-centric study includes 1.629 patients (Giessen: *n* = 786, Cologne: *n* = 843) with OPSCC, 1258 (77.2%) males and 371 (22.8%) females. All OPSCC were diagnosed between 1992 and 2020. Overall, the mean age at diagnosis was 59 years for both female and male patients (Fig. 1A and B). 311 (31.6%) male and 100 (34%) female patients had HPV-positive OPSCC (Fig. 1C and D, Table 1). The mean age of the patients with HPV-related OPSCC was 61 years, whereas in the HPV-negative OPSCC cohort it was 59 years. There was a significant difference in the distribution of the AJCC stages between men and women (*p* = 0.015, Fig. 1E and F, Table 1). 56.7% of the female patients were classified as T1-T2, whereas only 50.8% of the male patients were classified in this group (Table 1). Female patients demonstrated a trend for smaller tumor size (defined as T1-T2) compared to men (*p* = 0.055, Table 1). With regards to the lymph node status (N-stage of the TNM classification) and distant metastasis (M-stage of the classification), there was no significant difference between females and males (N-stage *p* = 0.512; M-stage *p* = 0.129, Table 1).

**Table 1:** Patient- and tumor-characteristics, risk factors and therapy modality according to gender

	(n) total cohort	%	(n) female	%	(n) male	%	p (male vs. female)
HPV-Status							
p16-HPV-	758	59.6	170	59.8	590	59.6	
p16-HPV +	40	3.2	9	3.1	31	3.2	
p16 + HPV-	62	4.9	7	2.4	55	5.6	
HPV- (total)	860	67.7	188	65.3	672	68.4	0.325
HPV +	411	32.3	100	34.7	311	31.6	
Unknown	358		83		275		
T-classification							
T1	311	20.1	78	22.7	233	19.4	0.294
T2	495	32.0	116	33.7	379	31.5	
T3	328	21.2	69	20.1	259	21.5	
T4	412	26.6	81	23.5	331	27.5	
Tx	83		27		56		
T1-T2	806	52.1	195	56.7	611	50.8	0.055
T3-T4	740	47.9	149	43.3	591	49.2	
N-classification							
N0	390	26.2	94	28.1	296	25.6	0.521
N1	226	15.2	56	16.7	170	14.7	
N2	775	52.0	165	49.3	610	52.8	
N3	100	6.7	20	6.0	80	6.9	
Nx	138		36		102		
M-classification							
M0	1369	94.2	309	96.0	1060	93.7	0.129
M1	84	5.8	13	4.0	71	6.3	
Mx	176		49		127		
Tumor stage (AJCC 8th edition)							
I	225	19.6	67	25.9	158	17.8	**0.015**
II	225	19.6	44	17.0	181	20.3	
III	195	17.0	48	18.5	147	16.5	
IV	504	43.9	100	38.6	404	45.4	
Not staged	480		112		368		
Smoking							
No smoking	307	26.2	67	26.7	240	26.0	0.832
Smoking	866	73.8	184	73.3	682	74.0	
Unknown	456		120		336		
Alcohol							
No alcohol	683	56.1	183	66.8	500	53.0	
Alcohol abuse	534	43.9	91	33.2	443	47.0	**< 0.001**
Unknown	412		97		315		
Treatment							
Surgery ± adj. therapy	897	65.8	210	69.5	687	64.8	0.122
Def. RCT	466	34.2	92	30.5	374	35.2	
unknown	266		69		197		
ECOG							
0	134	17.3	30	16.1	104	17.7	0.963
I	438	56.5	107	57.5	331	56.2	
II	166	21.4	39	21.0	127	21.6	
III	32	4.1	9	4.8	23	3.9	
IV	5	0.6	1	0.5	4	0.7	
Unknown	854		185		669		

%: percentage based on cases with known values. HPV + defined as p16 + /HPV + ; HPV- (total) defined as either p16-/HPV -, p16-/HPV + or p16 + /HPV-.Statistical analysis was done with the Chi Quadrat test, significant values in bolt

Of the female patients, 184 were smokers (73.3%) and 67 were non-smokers (26.7%). Of the male patients, 682 (74%) were smokers and 240 (26%) were non-smokers. There was no significant difference between males and females according to smoking (*p* = 0.832, Table 1, Fig. 1G, H). A higher number of male patients stated regular alcohol consumption compared to female patients (47% vs. 33.2%, *p* < 0.001, Table 1, Fig. 1I, J).

Regarding treatment modalities, 210 (69.5%) female patients received surgery (± adjuvant therapy) and 92 (30.5%) definitive RCT, whereas 687 (64.8%) of the male patients received surgery (± adjuvant therapy) and 374 (35.2%) were treated with definitive RCT (*p* = 0.122, Fig. 1K, L).

### Gender-specific overall survival

**Fig. 2:**
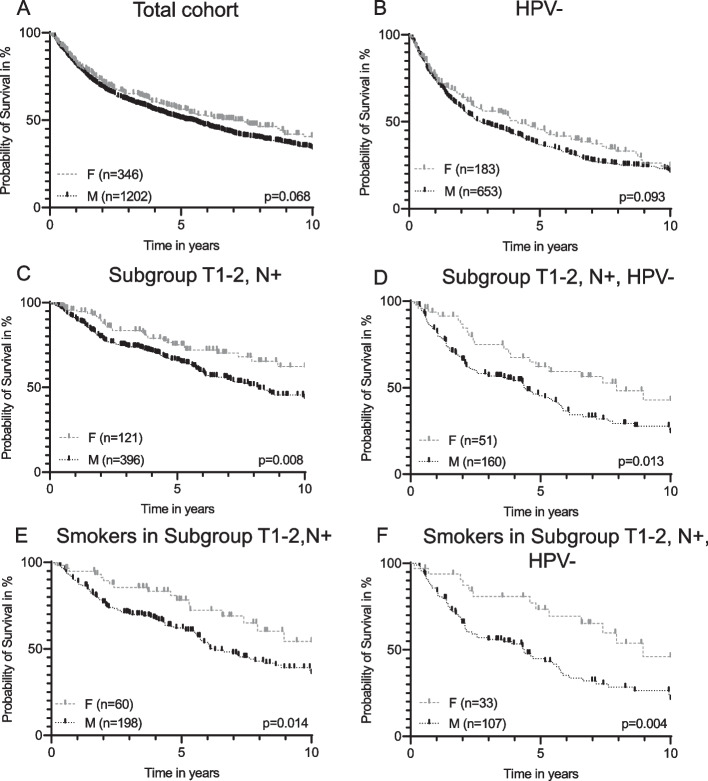
Gender-specific overall survival. Gender-specific overall survival **A** in the total cohort (*n* = 1548); **B** In patients with Human papillomavirus-negative (HPV-) OPSCC (HPV-negative defined as either p16-/HPV-, p16-/HPV + and p16 + /HPV; *n* = 836); **C** In subgroup T1-2, N + (*n* = 517); **D** in subgroup T1-2, N + , HPV- (*n* = 211); **E** In subgroup T1-2, N + , nicotine + , (*n* = 258); **F** In subgroup T1-2, N + , HPV-, nicotine + (*n* = 140)

A major objective of this study was to analyze the gender-specific OS of the total cohort and according to subgroups. Whereas gender itself could not be identified as a significant factor according to OS in the uni- and multivariate analysis (additional Table 1), there was a trend of better OS in female patients compared with male patients (*p* = 0.068, Fig. 2A) in the whole cohort. For men, the average survival time was 8.1 years, while for women it was 9.2 years. Similar to the total cohort, female patients with HPV-negative OPSCC demonstrated a trend for a better OS compared with males with HPV-negative OPSCC (*p* = 0.093, Fig. 2B). In patients with HPV-related OPSCC, there was no difference in OS in respect to sex (additional Fig. 1A).

We evaluated the OS in subgroups stratified by tumor size (T1-T2 vs. T3-T4) and with (N +) or without (N0) lymph node metastasis. In the subgroup with small tumors and lymph node metastasis (T1-T2, N +), women demonstrated a significant better OS than men (*p* = 0.008, Fig. 2C). Looking at the HPV-negative OPSCC in this subgroup, women also presented with a significant better OS (*p* = 0.013, Fig. 2D). In the other subgroups (T1-T2N0, T3-T4N + , T3-T4N0), as well as in the HPV-related cohort (additional Fig. 1B), there were no significant differences in OS between males and females.

Regarding OS analyzed according to the risk factors alcohol and smoking, the subgroup T1-T2, N + female smokers had a significantly improved OS compared with male smokers (*p* = 0.014, Fig. 2E). Similar to the results above, only in HPV-negative OPSCC with T1-T2, N + , female smokers demonstrated a significantly better OS compared with male smokers (*p* = 0.004, Fig. 2F). This was not evident in the cohort with HPV-related OPSCC (additional Fig. 1C). Whereas ECOG itself was a significant factor for OS in uni- and multivariate analysis (additional Table 1) in regard to sex there was no significant difference (*p* = 0.812, data not shown).

### Overall survival according to AJCC tumor staging, 8th edition

**Fig. 3:**
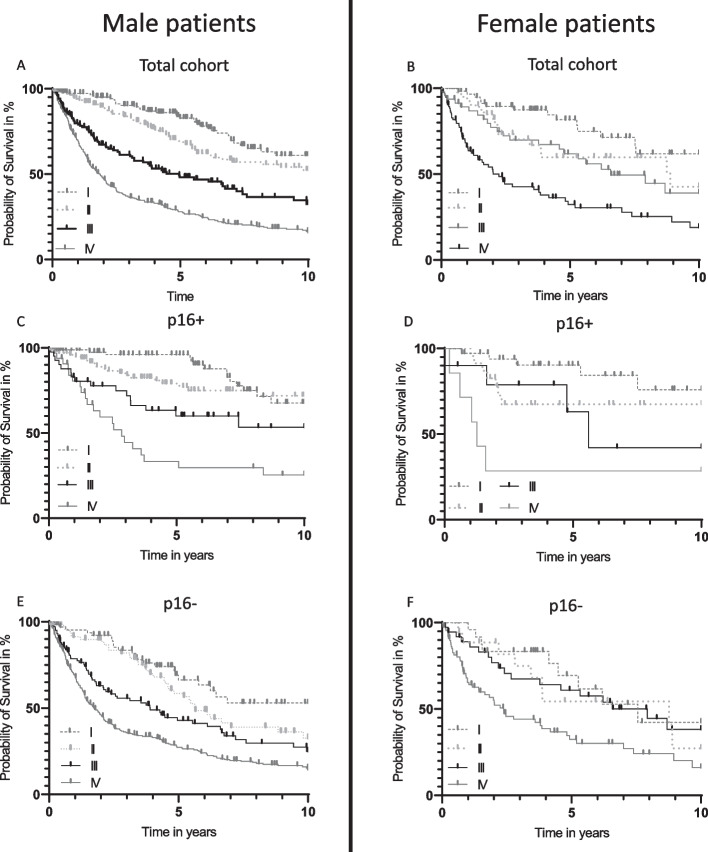
Overall survival according to AJCC-stage (American Joint Committee of Cancer 8th edition). Overall survival of **A** All male patients (*n* = 873, p(I vs. II) = 0.017, p(II vs. III) < 0.001, p(III vs. IV) < 0.001); **B** All female patients (*n* = 255, p(I vs. II) = 0.023, p(II vs. III) = 0.992, p(III vs. IV) = 0.001); **C** Male patients with p16-positive OPSCC (p16-positive defined as either p16 + /HPV- or p16 + HPV + ; *n* = 267, p(I vs. II) = 0.005, p(II vs. III) = 0.030, p(III vs. IV) = 0.004); **D** Female patients with p16-positive-OPSCC (*n* = 83, p(I vs. II) = 0.033, p(II vs. III) = 0.977, p(III vs. IV) = 0.090); **E** Male patients with p16-negative OPSCC (p16-negative defined as either p16-/HPV- or p16-HPV-; *n* = 605, p(I vs. II) = 0.437, p(II vs. III) = 0.008 p(III vs. IV) = 0.002); **F** Female patients with p16-negative OPSCC (*n* = 172, p(I vs. II) = 0.317, p(II vs. III) = 0.905, p(III vs. IV) = 0.005)

In the total cohort, male patients displayed significant stratification between all stages (all *p* < 0.05, Fig. 3A). In contrast, female patients were lacking significance between stage II and III (*p* (II vs. III) = 0.992; Fig. 3B). For additional analysis, patients were subdivided according to their p16-status (independent of HPV-DNA, as defined in the AJCC 8^th^ edition). Similar to the total cohort, male patients with p16 positive OPSCC displayed a significant stratification of all stages (all *p* < 0.05, Fig. 3C), whereas females demonstrated significance only between stage I and II (*p* = 0.03, Fig. 3D). In the p16-negative cohort, male patients demonstrated significance only between stage II and III and between stage III and IV (p(II vs. III) = 0.008, p(III vs. IV) = 0.002, Fig. 3E), whereas females displayed a significant difference only between stage III and IV (*p* = 0.005, Fig. 3F). Comparing the prognosis of female and male patients of the total cohort, or the p16-positive or -negative cohort according to the different AJCC8 stages I-IV, there were no significant differences (not shown).

### Gender-specific overall survival according to treatment modality

**Fig. 4:**
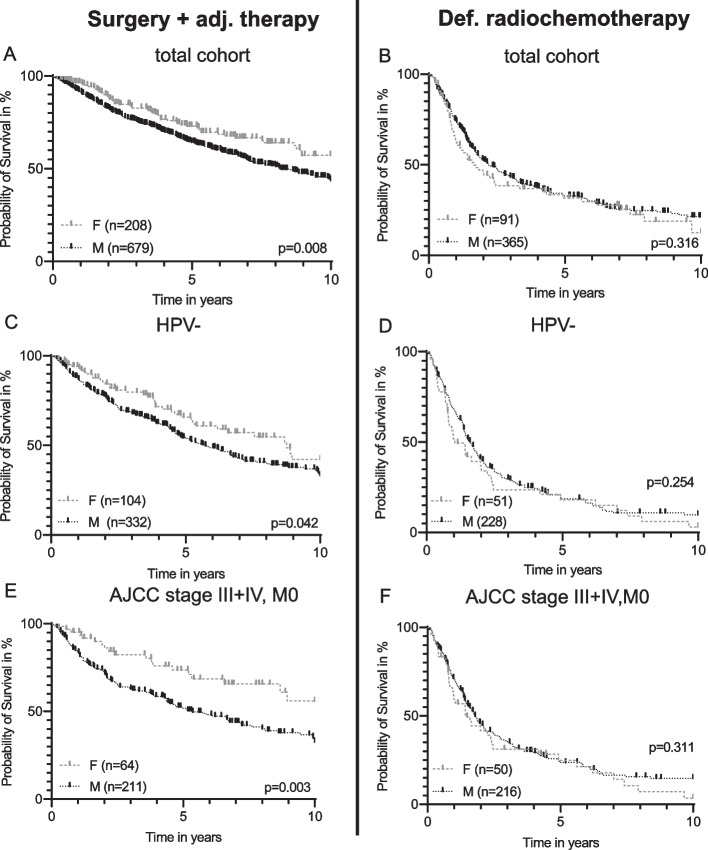
Gender specific overall survival according to therapy. Gender-specific overall survival of patients treated with surgery ± adjuvant therapy (**A**, **C**, **E**) or definitive radiochemotherapy (RCT) (**B**, **D**, **F**): **A** In the total cohort (*n* = 887); **B** In the total cohort (*n* = 456); **C** In the subgroup patients with Human papillomavirus-negative (HPV-) OPSCC (HPV-negative defined as either p16-/HPV-, p16-/HPV + or p16 + /HPV-; *n* = 436); **D** In the subgroup of patients with HPV- OPSCC (*n* = 279); **E** In the subgroup American Joint Committee of Cancer (AJCC) 8th edition stage III + IV,M0 (*n* = 275) **F** In the subgroup AJCC 8th edition III + IV, M0 (*n* = 266)

Female patients treated with surgery and risk-adapted adjuvant therapy had a significantly better OS compared to male patients with the same treatment (*p* = 0.008, Fig. 4A). In contrast, there was no significant difference between female and male patients when treated with def. RCT (*p* = 0.361, Fig. 4B).

When analyzing treatment modality according to HPV-status, female patients with HPV-negative OPSCC treated with surgery ± adjuvant therapy demonstrated a significantly better OS compared to male patients (*p* = 0.042, Fig. 4C). Similar to the results of the total cohort, no significant difference was seen in females with HPV-negative OPSCC treated with definitive RCT (*p* = 0.254, Fig. 4D). Patients with HPV-related OPSCC did not display any significant gender-specific differences according to both treatment options (additional Fig. 2A, B).

Finally, treatment modalities were examined in relation to the AJCC 8th edition. Similar to the results of the total cohort, we found that in advanced tumor stages (AJCC stage III, IV, M0) female patients treated with surgery ± adjuvant therapy had a significantly better OS (*p* = 0.003, Fig. 4E) compared to male patients. Further, there was no significant difference according to gender when treated with definitive RCT (*p* = 311, Fig. 4F).

## Discussion

Gender is an important aspect in oncology affecting incidence, treatment and prognosis. Nevertheless, there are still limited data on sex disparities in OPSCC, especially according to HPV-status. In this study, we could gain important insights into sex-related oncologic differences. First of all, we found that almost three times more men than women develop OPSCC, which is in line with data from literature [4, 37]. Various reasons are discussed like hormonal influence, immune response, HPV-status, but also a different lifestyle regarding sexual practices, tobacco and alcohol consumption [6, 14]. In our cohort, age, ECOG status, HPV-status, tumor size, lymph node- and distant metastasis as well as nicotine consumption were equally distributed between males and females. According to the equal distribution of the ECOG status, we can assume that patients were in a comparable state of health.

Furthermore, we could demonstrate that there is a trend for better OS in women compared with men. This is in line with a recently published study of Preissner et al. demonstrating a significantly better 5-year-OS of women in a cohort of almost 150.000 patients with HNSCC [14]. In addition to Preissner et al., we analyzed the OS in different subgroups. Thereby, we could identify that in the subgroup with small tumors and lymphatic metastasis (T1-T2, N +), female patients demonstrated a significantly better OS than men independent of smoking and alcohol consumption. We could also demonstrate that female patients treated with surgery ± adjuvant therapy vs. definitive RCT had a significantly better OS than men. The same results were found in female patients with advanced tumor stage (subgroup AJCC III-IV, M0).

Up-to-date there are no reliable data that adequately explains differences in OS between male and female patients with OPSCC [14]. Looking at other cancer entities, survival advantage of women is often explained with younger age at first diagnosis, less nicotine consumption or with distinct phenotypes of diseases by sex [38, 39].

Nicotine and alcohol consumption are well-known risk factors [13]. Regarding the risk factor smoking, we couldn’t detect gender-specific differences in the overall distribution. Therefore, this does not explain the better OS of women in our OPSCC cohort. One limitation thereby is the lack of granularity with regard to smoking history, which is considered binary because sufficient pack-year data were not available. However, we could demonstrate that smoking seems to have an effect on OS in males and females within certain subgroups: Smoking female presented with a significantly better OS in the subgroup of T1-T2, N + OPSCC compared with males. This can’t be explained by different treatments of males and females as both displayed equal distribution to surgical therapy vs. definitive RCT (Table 1). One explanation might be that smoking affects hormone balance differently in women than in men, as smoking is known to increase estrogen catabolism [36] and thus could confer a survival advantage over men.

Furthermore, we found that significantly more males than females reported alcohol consumption in the total cohort. Alcohol consumption is an important risk factor especially in HPV-negative OPSCC. We therefore analyzed the influence of alcohol consumption also in T1-T2, N + OPSCC and in the subgroup T1-T2, N + , HPV-negative OPSCC (not shown). Again, no significant difference was found between men and women, so the risk factor of alcohol could not explain the better OS of women. These findings are in line with the results of Preissner et al. [14], who could not detect differences in tobacco and alcohol consumption according to sex in their large study population of HNSCC. A limitation of our study is, that we weren’t able to assess the quantity of alcohol consumption due to insufficient data available over the years.

Besides smoking and alcohol, HPV-status is the most important risk- and prognostic factor in OPSCC patients to date. In line with other representative studies such as the RTOG-0129 [13], our study revealed a significantly better 5-year & 10-year OS of patients with HPV-related OPSCC compared to patients with HPV-negative OPSCC (additional Fig. 3A, B). In our study cohort, HPV positivity was defined as a combination of HPV-DNA and p16 positivity. This is important to emphasize, as the definition of HPV positive OPSCC (whether it is defined only by overexpression of p16, independent of HPV-DNA or a combination of HPV-DNA and p16 positivity) is still discussed controversially in literature and not uniform [9, 37, 40–42]. In addition to the p16 + /HPV + cases we could identify in total 62 patients with p16 overexpression but HPV-DNA negative OPSCC (which is a rate of 4.9% of false positive p16 cases).This rate is comparable with the predicted rate of false positive p16 cases (3.8%) calculated by a recently published formula of Gallus et al. [42]. Recent studies [9, 37, 40–42] found that patients with p16 + /HPV- OPSCC seem to have the same prognosis or only slightly better than p16-/HPV- cases. Consistent with these findings patients in our cohort with p16 + /HPV- OPSCC (and p16-/HPV + OPSCC) had a significant worse OS than patients with p16 + /HPV + OPSCC (additional Fig. 3C & D).

Interestingly, when OS was analyzed with respect to gender, we could demonstrate better OS of women only in the HPV-negative cohort. This finding contradicts the results of Preissner et al. [14] where better OS of women was found in the HPV-related cohort. Mentioned by the authors themselves, one weakness in their retrospective study cohort was the unclear origin of the HPV test samples. In our study cohort, HPV positivity was defined as a combination of HPV-DNA and p16 positivity, which might in parts explain some discordance of the results. A limitation of our study is the missing investigation on the disease-free survival (DFS), due to the retrospective study design and lacking data on cause of death in most cases. This would have given more accurate information on survival of OPSCC patients based on the disease itself.

Importantly, in our study there were no gender-specific differences in the distribution of HPV-status. Consequently, HPV-status of OPSCC affected the survival probability equally and therefore could not explain the gender-specific differences in OS.

Regarding AJCC8 stages, male patients demonstrated a significant stratification between all stages, whereas women were lacking significance between stage II and III. We could further demonstrate that male patients with p16 negative OPSCC, as well as females with p16 positive and negative OPSCC were lacking significance between different stages. However, this aspect needs to be reviewed within a larger cohort.

## Conclusion

In summary, we could demonstrate important sex-specific differences: Females had significantly better OS than males in the subgroup T1-2, N + , independent of risk factors. Men demonstrated significantly better stratification across all AJCC8 stages. In contrast, women did not reveal OS differences between stage II vs. stage III. A comparison of therapy (surgery ± adjuvant therapy vs definitive RCT) demonstrated, that women treated with surgery ± adjuvant therapy had better OS than men in the whole cohort. The same results were detected in the cohort of HPV-negative OPSCC and within high-risk groups (AJCC8 stage III and IV with M0). The cause for these gender disparities cannot be explained adequately by our study and needs to be addressed in the future. Understanding gender differences in OS in OPSCC could impact future treatment strategies, especially in the era of personalized medicine.

### Supplementary Information

**Additional file 1:** **Supplement Table 1.** Univariate and multivariate survival analysis according to risk factors and tumor characteristics in the whole cohort (*n* =1629). **Supplement Table 2.** Univariate and multivariate survival analysis according to risk factors and tumor characteristics in the female cohort (*n* =371). **Supplement Table 3.** Univariate and multivariate survival analysis according to risk factors and tumor characteristics in the male cohort (*n* =1258).

**Additional file 2.** Gender-specific overall survival of Human papillomavirus-positive patients: Gender-specific overall survival A In Human papillomavirus-positive (HPV+) patients (defined as p16+/HPV+; *n* = 403); B In subgroup T1-2, N+, HPV+ (*n* = 203); C In subgroup T1-2, N+, HPV+, nicotine+ (*n* = 72).

**Additional file 3. **Gender specific overall survival according to therapy. Gender-specific overall survival of patients treated with surgery +/- adjuvant therapy (A, C) or definitive radiochemotherapy (RCT) (B, D). A In the subgroup p16-positive (p16+) patients (defined as either p16+/HPV+ or p16+/HPV-; *n* = 247); B In the subgroup p16+ patients (*n* = 109), C In the subgroup American Joint Committee of Cancer (AJCC)8th edition stage I+II (*n* = 369); D In the subgroup AJCC8th I+II (*n* = 43).

**Additional file 4.** Overall Survival in Human papillomavirus positive vs. Human papillomavirus negative cohort. A In the male cohort (*n* = 957); B In the female cohort (*n* = 281); C + D In the total cohort (*n*=1238). HPV-negative defined as either p16-/HPV-, p16-/HPV+ and p16+/HPV-; HPV-positive defined as p16+/HPV+; ** = p.

## Data Availability

With regard to potentially personalized data, ethical, and legal rules, data can be made available upon reasonable request from the corresponding author for academic research within the constraints of the consent given by the patients.

## Abbreviations

AJCC: American Joint Committee of Cancer
CI: Confidence Intervall
Def.: Definitive
HNSCC: Head and Neck Squamous Cell Carcinoma
HPV: Human Papilloma Virus
HR: Hazard Ratio
PCR: Polymerase Chain Reaction
n.a.: Not applicable
OPSCC: Oropharyngeal Squamous Cell Carcinoma
OS: Overall Survival
R(C)T: Radio(chemo)therapy

## References

[1] Sung H, Ferlay J, Siegel RL, Laversanne M, Soerjomataram I, Jemal A (2021). Global cancer statistics 2020: GLOBOCAN estimates of incidence and mortality worldwide for 36 cancers in 185 countries. CA Cancer J Clin.

[2] Chaturvedi AK, Graubard BI, Broutian T, Pickard RKL, Tong Z, Xiao W (2015). NHANES 2009–2012 findings: association of sexual behaviors with higher prevalence of oral oncogenic human papillomavirus infections in U.S. men. Cancer Res.

[3] Lechner M, Liu J, Masterson L, Fenton TR (2022). HPV-associated oropharyngeal cancer: epidemiology, molecular biology and clinical management. Nat Rev Clin Oncol.

[4] Wittekindt C, Wagner S, Bushnak A, Prigge E-S, von Knebel DM, Würdemann N (2019). Increasing Incidence rates of oropharyngeal squamous cell carcinoma in Germany and significance of disease burden attributed to human papillomavirus. Cancer Prev Res (Phila Pa).

[5] Jethwa AR, Khariwala SS (2017). Tobacco-related carcinogenesis in head and neck cancer. Cancer Metastasis Rev.

[6] Hashibe M, Brennan P, Chuang S, Boccia S, Castellsague X, Chen C (2009). Interaction between Tobacco and alcohol use and the risk of head and neck cancer: pooled analysis in the international head and neck cancer epidemiology consortium. Cancer Epidemiol Biomarkers Prev.

[7] Chaturvedi AK, Engels EA, Anderson WF, Gillison ML (2008). Incidence trends for human papillomavirus-related and –unrelated oral squamous cell carcinomas in the United States. J Clin Oncol.

[8] Carlander A-LF, Grønhøj Larsen C, Jensen DH, Garnæs E, Kiss K, Andersen L (2017). Continuing rise in oropharyngeal cancer in a high HPV prevalence area: a Danish population-based study from 2011 to 2014. Eur J Cancer.

[9] Wagner S, Prigge E-S, Wuerdemann N, Reder H, Bushnak A, Sharma SJ (2020). Evaluation of p16(INK4a) expression as a single marker to select patients with HPV-driven oropharyngeal cancers for treatment de-escalation. Br J Cancer.

[10] Kreimer AR, Clifford GM, Boyle P, Franceschi S (2005). Human papillomavirus types in head and neck squamous cell carcinomas worldwide: a systematic review. Cancer Epidemiol Biomarkers Prev.

[11] Klussmann JP, Gültekin E, Weissenborn SJ, Wieland U, Dries V, Dienes HP (2003). Expression of p16 protein identifies a distinct entity of tonsillar carcinomas associated with human papillomavirus. Am J Pathol.

[12] Fakhry C, Westra WH, Li S, Cmelak A, Ridge JA, Pinto H (2008). Improved survival of patients with human papillomavirus-positive head and neck squamous cell carcinoma in a prospective clinical trial. JNCI J Natl Cancer Inst.

[13] Ang KK, Harris J, Wheeler R, Weber R, Rosenthal DI, Nguyen-Tân PF (2010). Human papillomavirus and survival of patients with oropharyngeal cancer. N Engl J Med.

[14] Preissner SH, Nahles S, Preissner S, Heiland M, Koerdt S (2022). Influence of sex on survival rates of HPV-positive oropharyngeal cancers. Front Oncol.

[15] Castellsagué X, Alemany L, Quer M, Halec G, Quirós B, Tous S (2016). HPV involvement in head and neck cancers: comprehensive assessment of biomarkers in 3680 patients. JNCI J Natl Cancer Inst.

[16] Cole L, Polfus L, Peters ES (2012). Examining the incidence of human papillomavirus-associated head and neck cancers by race and ethnicity in the U.S., 1995–2005. PLOS ONE.

[17] Quaas A, Biesma HD, Wagner AD, Verheij M, van Berge Henegouwen MI, Schoemig-Markiefka B (2022). Microsatellite instability and sex differences in resectable gastric cancer – a pooled analysis of three European cohorts. Eur J Cancer.

[18] Rahbari R, Zhang L, Kebebew E (2010). Thyroid cancer gender disparity. Future Oncol Lond Engl.

[19] Wagner AD, Oertelt-Prigione S, Adjei A, Buclin T, Cristina V, Csajka C (2019). Gender medicine and oncology: report and consensus of an ESMO workshop. Cancer-Relat Cogn Impair.

[20] Yuan Y, Liu L, Chen H, Wang Y, Xu Y, Mao H (2016). Comprehensive characterization of molecular differences in cancer between male and female patients. Cancer Cell.

[21] Auguste A, Joachim C, Deloumeaux J, Gaete S, Michineau L, Herrmann-Storck C (2021). Head and neck cancer risk factors in the French West Indies. BMC Cancer.

[22] Usyk M, Zolnik CP, Castle PE, Porras C, Herrero R, Gradissimo A (2020). Cervicovaginal microbiome and natural history of HPV in a longitudinal study. PLOS Pathog.

[23] KombeKombe AJ, Li B, Zahid A, Mengist HM, Bounda G-A, Zhou Y (2021). Epidemiology and burden of human papillomavirus and related diseases, molecular pathogenesis, and vaccine evaluation. Front Public Health.

[24] Hernandez BY, Wilkens LR, Zhu X, Thompson P, McDuffie K, Shvetsov YB (2008). Transmission of human papillomavirus in heterosexual couples. Emerg Infect Dis J.

[25] Widdice L, Ma Y, Jonte J, Farhat S, Breland D, Shiboski S (2013). Concordance and transmission of human papillomavirus within heterosexual couples observed over short intervals. J Infect Dis.

[26] Saunders CL, Meads C, Abel GA, Lyratzopoulos G (2017). Associations between sexual orientation and overall and site-specific diagnosis of cancer: evidence from two national patient surveys in England. J Clin Oncol.

[27] D’Souza G, Cullen K, Bowie J, Thorpe R, Fakhry C (2014). Differences in oral sexual behaviors by gender, age, and race explain observed differences in prevalence of oral human papillomavirus infection. PLoS ONE.

[28] Chung CH, Bagheri A, D’Souza G (2014). Epidemiology of oral human papillomavirus infection. Oral Oncol.

[29] Morand GB, Cardona I, Cruz SB, Mlynarek AM, Hier MP, Alaoui-Jamali MA (2022). Therapeutic vaccines for HPV-associated oropharyngeal and cervical cancer: the next de-intensification strategy?. Int J Mol Sci.

[30] Sudenga SL, Torres BN, Silva R, Villa LL, Lazcano-Ponce E, Abrahamsen M (2017). Comparison of the Natural history of genital HPV infection among men by Country: Brazil, Mexico, and the United States. Cancer Epidemiol Biomarkers Prev.

[31] Darwich L, Cañadas M-P, Videla S, Coll J, Molina-López RA, Sirera G (2013). Prevalence, clearance, and incidence of human papillomavirus type-specific infection at the anal and penile site of HIV-infected men. Sex Transm Dis.

[32] Chow EPF, Tabrizi SN, Fairley CK, Wigan R, Machalek DA, Garland SM (2021). Prevalence of human papillomavirus in young men who have sex with men after the implementation of gender-neutral HPV vaccination: a repeated cross-sectional study. Lancet Infect Dis.

[33] Rollo F, Latini A, Pichi B, Colafigli M, Benevolo M, Sinopoli I (2017). Prevalence and determinants of oral infection by Human Papillomavirus in HIV-infected and uninfected men who have sex with men. PLoS ONE.

[34] Naugler WE, Sakurai T, Kim S, Maeda S, Kim K, Elsharkawy AM (2007). Gender disparity in liver cancer due to sex differences in MyD88-dependent IL-6 production. Science.

[35] Hashim D, Sartori S, Vecchia CL, Serraino D, Maso LD, Negri E (2017). Hormone factors play a favorable role in female head and neck cancer risk. Cancer Med.

[36] Rosenberg M, Waugh M, Stevens C (1996). Smoking and cycle control among oral contraceptive users. Am J Obstet Gynecol.

[37] Mehanna H, Taberna M, von Buchwald C, Tous S, Brooks J, Mena M, et al. Prognostic implications of p16 and HPV discordance in oropharyngeal cancer (HNCIG-EPIC-OPC): a multicentre, multinational, individual patient data analysis. Lancet Oncol n.d. 10.1016/S1470-2045(23)00013-X. 10.1016/S1470-2045(23)00013-X 36796393

[38] Micheli A, Ciampichini R, Oberaigner W, Ciccolallo L, de Vries E, Izarzugaza I (2009). The advantage of women in cancer survival: an analysis of EUROCARE-4 data. Eur J Cancer.

[39] Wakelee HA, Wang W, Schiller JH, Langer CJ, Sandler AB, Belani CP (2006). Survival differences by sex for patients with advanced non-small cell lung cancer on Eastern cooperative oncology group trial 1594. J Thorac Oncol.

[40] Nauta I, Rietbergen M, Bokhoven A, Bloemena E, Witte B, Heideman D (2018). Evaluation of the 8th TNM classification on p16-positive oropharyngeal squamous cell carcinomas in the Netherlands, and the importance of additional HPV DNA-testing. Ann Oncol.

[41] Bussu F, Sali M, Gallus R, Vellone VG, Zannoni GF, Autorino R (2013). HPV infection in squamous cell carcinomas arising from different mucosal sites of the head and neck region. Is p16 immunohistochemistry a reliable surrogate marker?. Br J Cancer.

[42] Gallus R, Nauta IH, Marklund L, Rizzo D, Crescio C, Mureddu L (2023). Accuracy of p16 IHC in classifying HPV-driven OPSCC in different populations. Cancers.

