# Supplementary material for: Sex-specific aspects in patients with oropharyngeal squamous cell carcinoma: a bicentric cohort study
Source: BMC Cancer. 2023 Nov 2;23:1054. doi: 10.1186/s12885-023-11526-6 (PMC10621233; doi:10.1186/s12885-023-11526-6)
Supplement: Supplementary file 1 — Additional file 1: Supplement Table 1. Univariate and multivariate survival analysis according to risk factors and tumor characteristics in the whole cohort (n =1629). Supplement Table 2. Univariate and multivariate survival analysis according to risk factors and tumor characteristics in the female cohort (n =371). Supplement Table 3. Univariate and multivariate survival analysis according to risk factors and tumor characteristics in the male cohort (n =1258). [file 12885_2023_11526_MOESM1_ESM.docx]

Supplement table 1: Univariate and multivariate survival analysis according to risk factors and tumor characteristics in the whole cohort (n =1629)

|  | **Univariate**  **Median Survival in Years** | | | | | | | **Multivariate** | | | | | | | |
| --- | --- | --- | --- | --- | --- | --- | --- | --- | --- | --- | --- | --- | --- | --- | --- |
|  |  |  |  |  |  |  |  | ***all factors included*** | | | | ***only significant factors included*** | | | |
|  |  | **N** | **OS** | **CI** | | **p** | **10y-OS** | **HR** | **CI** | | **p^a^** | **HR** | **CI** | | **p^a^** |
|  |  |  |  | **Lower** | **Upper** |  |  |  | **Lower** | **Upper** |  |  | **Lower** | **Upper** |  |
| **Age** | <60 years | 781 | 8.821 | 6.401 | 8.821 | **<0.001** | 57.1% | 1.677 | 1.267 | 2.220 | **<0.001** | 1.686 | 1.279 | 2.222 | **<0.001** |
|  | >60 years | 771 | 4.640 | 3.317 | 4.640 |  | 48.1% |  |  |  |  |  |  |  |  |
| **Sex** | female | 371 | 7.008 | 4.957 | 9.060 | 0.068 | 58.1% | 0.975 | 0.704 | 1.350 | 0.880 |  |  |  |  |
|  | male | 1258 | 5.211 | 4.482 | 5.940 |  | 51.0% |  |  |  |  |  |  |  |  |
| **HPV** | HPV negative | 860 | 3.063 | 2.524 | 3.602 | **<0.001** | 38.6% | 0.393 | 0.251 | 0.615 | **<0.001** | 0.419 | 0.278 | 0.630 | **<0.001** |
|  | HPV positive | 411 | n.a. | n.a. | n.a. |  | 71.4% |  |  |  |  |  |  |  |  |
| **T Status** | T1-2 | 806 | 8.884 | n.a. | n.a. | **<0.001** | 64.4% | 1.778 | 1.270 | 2.489 | **<0.001** | 1.777 | 1.270 | 2.486 | **<0.001** |
|  | T3-4 | 740 | 2.460 | 2.038 | 2.882 |  | 39.6% |  |  |  |  |  |  |  |  |
| **N Status** | N0 | 390 | 7.200 | 5.633 | 8.767 | **<0.001** | 59.4% | 1.185 | 0.854 | 1.644 | 0.310 |  |  |  |  |
|  | N+ | 1101 | 5.129 | 4.386 | 5.871 |  | 50.1% |  |  |  |  |  |  |  |  |
| **ECOG** | 0-1 | 572 | 6.101 | 4.881 | 7.322 | **<0.001** | 57.5% | 1.815 | 1.352 | 2.436 | **<0.001** | 1.831 | 1.367 | 2.453 | **<0.001** |
|  | 2-4 | 197 | 1.411 | 0.996 | 1.826 |  | 24.7% |  |  |  |  |  |  |  |  |
| **Therapy** | Surgery | 897 | 8.945 | n.a | n.a. | **<0.001** | 63.7% | 1.782 | 1.267 | 2.507 | **<0.001** | 1.878 | 1.346 | 2.620 | **<0.001** |
|  | Def. RCT | 466 | 2.170 | 1.750 | 2.589 |  | 38.4% |  |  |  |  |  |  |  |  |
| **Alcohol** | no | 683 | 8.884 | n.a. | n.a. | **<0.001** | 62.2% | 1.439 | 1.077 | 1.922 | **0.014** | 1.449 | 1.090 | 1.927 | **0.011** |
|  | yes | 534 | 3.858 | 3.079 | 4.636 |  | 40.5% |  |  |  |  |  |  |  |  |
| **Smoking** | No | 307 | 8.526 | n.a. | n.a. | **0.002** | 60.4% | 0.902 | 0.591 | 1.377 | 0.633 |  |  |  |  |
|  | yes | 866 | 5.129 | 4.388 | 5.869 |  | 46.6% |  |  |  |  |  |  |  |  |

OS: Overal Survival: HR: hazard ratios estimated by Cox proportional-hazards models; CI: 95% confidence interval. p-values calculated by Log Rank (Mantel–Cox) test; univariate; p < 0.05 in bold; ^a^ p-values estimated by Cox proportional-hazards models, uni- and multivariate; p < 0.05 in bold, n.a. : not applicable

Supplement table 2: Univariate and multivariate survival analysis according to risk factors and tumor characteristics in the female cohort (n =371)

|  | **Univariate**  **Median Survival in Years** | | | | | | | **Multivariate** | | | | | | | |
| --- | --- | --- | --- | --- | --- | --- | --- | --- | --- | --- | --- | --- | --- | --- | --- |
|  |  |  |  |  |  |  |  | ***all factors included*** | | | | ***only significant factors included*** | | | |
|  |  | **N** | **OS** | **CI** | | **p** | **10y-OS** | **HR** | **CI** | | **p^a^** | **HR** | **CI** | | **p^a^** |
|  |  |  |  | **Lower** | **Upper** |  |  |  | **Lower** | **Upper** |  |  | **Lower** | **Upper** |  |
| **Age** | <60 years | 170 | 8.684 | n.a. | n.a. | **<0.001** | 59.4% | 1.157 | 0.585 | 2.289 | 0.675 |  |  |  |  |
|  | >60 years | 176 | 6.568 | 4.415 | 8.721 |  | 56.8% |  |  |  |  |  |  |  |  |
| **HPV** | HPV negative | 188 | 4.123 | 2.813 | 5.434 | **<0.001** | 45.4% | 0.270 | 0.097 | 0.750 | **0.012** | 0.293 | 0.131 | 0.656 | **0.003** |
|  | HPV positive | 100 | n.a. | n.a. | n.a. |  | 72.4% |  |  |  |  |  |  |  |  |
| **T Status** | T1-2 | 195 | n.a. | n.a. | n.a. | **<0.001** | 72.7% | 2.166 | 0.958 | 4.897 | **0.063** | 2.446 | 1.148 | 5.212 | **0.021** |
|  | T3-4 | 149 | 2.170 | 1.256 | 3.083 |  | 39.6% |  |  |  |  |  |  |  |  |
| **N Status** | N0 | 94 | 7.551 | 4.807 | 10.294 | **<0.001** | 61.7% | 0.952 | 0.491 | 1.846 | 0.885 |  |  |  |  |
|  | N+ | 241 | 7.008 | 4.200 | 9.816 |  | 57.0% |  |  |  |  |  |  |  |  |
| **ECOG** | 0-1 | 137 | 7.926 | 5.165 | 10.687 | **<0.001** | 64.2 % | 1.169 | 0.578 | 2.364 | 0.663 |  |  |  |  |
|  | 2-4 | 49 | 1.167 | 0.268 | 2.066 |  | 24.5% |  |  |  |  |  |  |  |  |
| **Therapy** | Surgery | 210 | n.a. | n.a. | n.a. | **<0.001** | 72.1% | 2.929 | 1.292 | 6.640 | **0.010** | 3.066 | 1.443 | 6.516 | **0.004** |
|  | Def. RCT | 92 | 1.649 | 1.038 | 2.261 |  | 36.3% |  |  |  |  |  |  |  |  |
| **Alcohol** | No | 183 | 9.659 | n.a. | n.a. | **<0.001** | 66.7% | 1.639 | 0.772 | 3.480 | 0.198 |  |  |  |  |
|  | Yes | 91 | 4.175 | 2.798 | 5.553 |  | 39.6% |  |  |  |  |  |  |  |  |
| **Smoking** | no | 67 | 9.659 | n.a. | n.a. | **0.002** | 61.2% | 0.498 | 0.200 | 1.238 | 0.133 |  |  |  |  |
|  | yes | 184 | 6.186 | 4.431 | 7.941 |  | 44.4% |  |  |  |  |  |  |  |  |

OS: Overal Survival: HR: hazard ratios estimated by Cox proportional-hazards models; CI: 95% confidence interval. p-values calculated by Log Rank (Mantel–Cox) test; univariate; p < 0.05 in bold; ^a^ p-values estimated by Cox proportional-hazards models, uni- and multivariate; p < 0.05 in bold, n.a. : not applicable

Supplement table 3: Univariate and multivariate survival analysis according to risk factors and tumor characteristics in the male cohort (n =1258)

|  | **Univariate**  **Median Survival in Years** | | | | | | | **Multivariate** | | | | | | | |
| --- | --- | --- | --- | --- | --- | --- | --- | --- | --- | --- | --- | --- | --- | --- | --- |
|  |  |  |  |  |  |  |  | ***all factors included*** | | | | ***only significant factors included*** | | | |
|  |  | **N** | **OS** | **CI** | | **p** | **10y-OS** | **HR** | **CI** | | **p^a^** | **HR** | **CI** | | **p^a^** |
|  |  |  |  | **Lower** | **Upper** |  |  |  | **Lower** | **Upper** |  |  | **Lower** | **Upper** |  |
| **Age** | <60 years | 611 | 7.365 | 6.022 | 8.708 | **<0.001** | 56.5% | 1.745 | 1.271 | 2.395 | **<0.001** | 1.743 | 1.276 | 2.381 | **<0.001** |
|  | >60 years | 591 | 3.674 | 2.955 | 4.393 |  | 45.3% |  |  |  |  |  |  |  |  |
| **HPV** | HPV negative | 672 | 5.608 | 3.776 | 7.441 | **<0.001** | 36.8% | 0.425 | 0.257 | 0.704 | **<0.001** | 0.428 | 0.269 | 0.680 | **<0.001** |
|  | HPV positive | 311 | n.a | n.a. | n.a. |  | 71.1% |  |  |  |  |  |  |  |  |
| **T Status** | T1-2 | 611 | 8.159 | 6.682 | 9.636 | **<0.001** | 61.7% | 1.634 | 1.124 | 2.375 | **0.010** | 1.684 | 1.158 | 2.448 | **0.006** |
|  | T3-4 | 591 | 2.490 | 2.054 | 2.927 |  | 39.7% |  |  |  |  |  |  |  |  |
| **N Status** | N0 | 290 | 7.009 | 4.813 | 9.205 | **<0.001** | 58.8% | 1.312 | 0.891 | 1.930 | 0.169 |  |  |  |  |
|  | N+ | 860 | 4.701 | 3.869 | 5.534 |  | 48.2% |  |  |  |  |  |  |  |  |
| **ECOG** | 0-1 | 435 | 5.723 | 4.944 | 6.502 | **<0.001** | 55.3% | 1.916 | 1.378 | 2.664 | **<0.001** | 1.920 | 1.380 | 2.671 | **<0.001** |
|  | 2-4 | 154 | 1.422 | 0.944 | 1.900 |  | 24.8% |  |  |  |  |  |  |  |  |
| **Therapy** | Surgery | 687 | 8.159 | 6.798 | 9.519 | **<0.001** | 61.3% | 1.607 | 1.100 | 2.348 | **0.014** | 1.718 | 1.186 | 2.488 | **0.004** |
|  | Def. RCT | 374 | 2.241 | 1.752 | 2.730 |  | 39.0% |  |  |  |  |  |  |  |  |
| **Alcohol** | no | 315 | 8.405 | n.a. | n.a. | **<0.001** | 60.6% | 1.401 | 1.018 | 1.930 | **0.039** | 1.415 | 1.029 | 1.945 | **0.033** |
|  | Yes | 443 | 3.805 | 2.898 | 4.713 |  | 40.7% |  |  |  |  |  |  |  |  |
| **Smoking** | no | 240 | 8.405 | n.a. | n.a. | **0.002** | 60.2% | 1.093 | 0.678 | 1.762 | 0.714 |  |  |  |  |
|  | yes | 682 | 4.625 | 3.863 | 5.387 |  | 44.4% |  |  |  |  |  |  |  |  |

OS: Overal Survival: HR: hazard ratios estimated by Cox proportional-hazards models; CI: 95% confidence interval. p-values calculated by Log Rank (Mantel–Cox) test; univariate; p < 0.05 in bold; ^a^ p-values estimated by Cox proportional-hazards models, uni- and multivariate; p < 0.05 in bold, n.a. : not applicable
