# Supplementary material for: Sex-specific aspects in patients with oropharyngeal squamous cell carcinoma: a bicentric cohort study
Source: BMC Cancer. 2023 Nov 2;23:1054. doi: 10.1186/s12885-023-11526-6 (PMC10621233; doi:10.1186/s12885-023-11526-6)
Supplement: Supplementary file 2 — Additional file 2. Gender-specific overall survival of Human papillomavirus-positive patients: Gender-specific overall survival A In Human papillomavirus-positive (HPV+) patients (defined as p16+/HPV+; n = 403); B In subgroup T1-2, N+, HPV+ (n = 203); C In subgroup T1-2, N+, HPV+, nicotine+ (n = 72). [file 12885_2023_11526_MOESM2_ESM.pptx]

## Slide 1
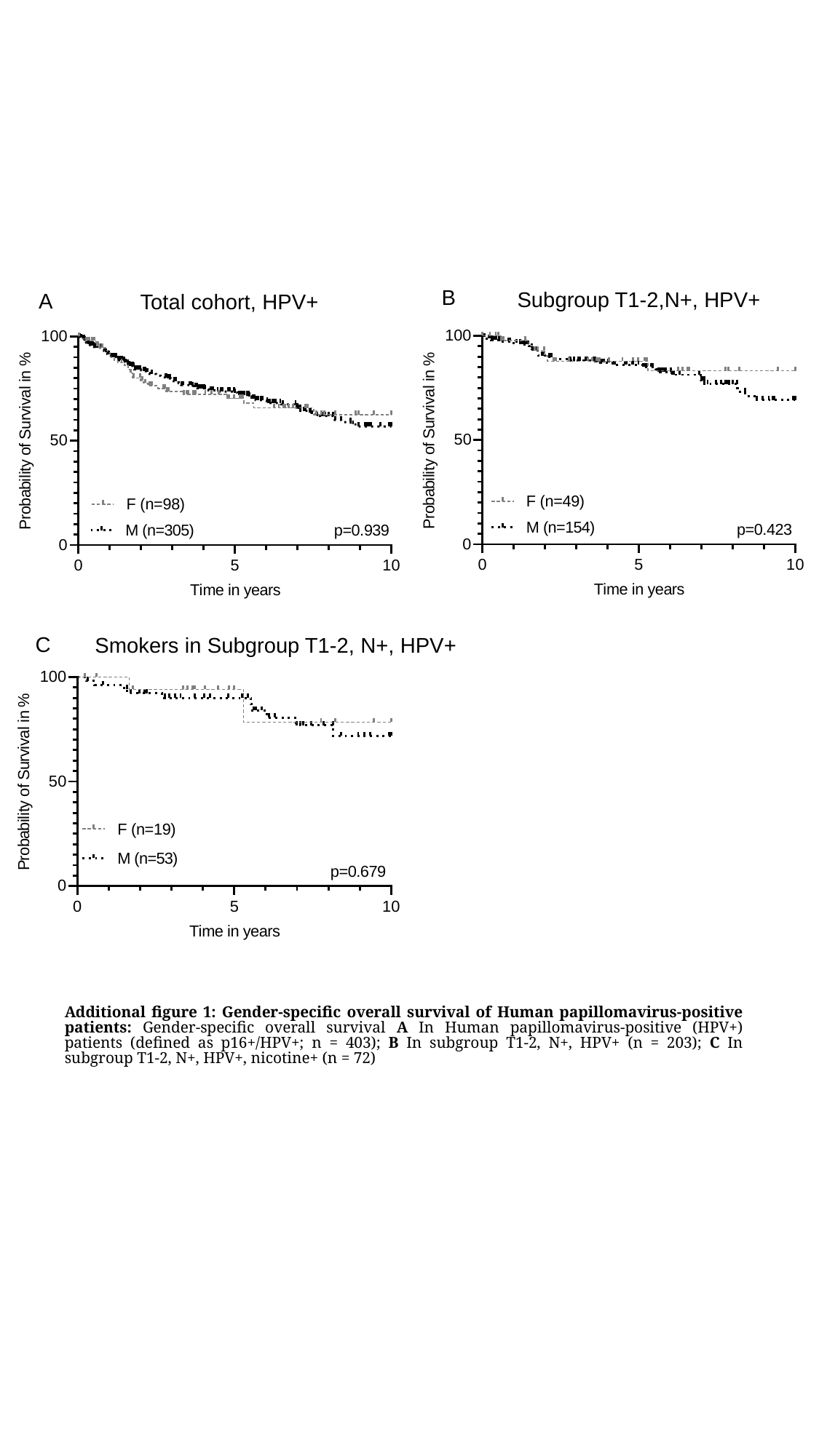

B
Subgroup T1-2,N+, HPV+
A
Total cohort, HPV+
C
Smokers in Subgroup T1-2, N+, HPV+
Additional figure 1: Gender-specific overall survival of Human papillomavirus-positive patients: Gender-specific overall survival A In Human papillomavirus-positive (HPV+) patients (defined as p16+/HPV+; n = 403); B In subgroup T1-2, N+, HPV+ (n = 203); C In subgroup T1-2, N+, HPV+, nicotine+ (n = 72)
