# Supplementary material for: Sex-specific aspects in patients with oropharyngeal squamous cell carcinoma: a bicentric cohort study
Source: BMC Cancer. 2023 Nov 2;23:1054. doi: 10.1186/s12885-023-11526-6 (PMC10621233; doi:10.1186/s12885-023-11526-6)
Supplement: Supplementary file 3 — Additional file 3. Gender specific overall survival according to therapy. Gender-specific overall survival of patients treated with surgery +/- adjuvant therapy (A, C) or definitive radiochemotherapy (RCT) (B, D). A In the subgroup p16-positive (p16+) patients (defined as either p16+/HPV+ or p16+/HPV-; n = 247); B In the subgroup p16+ patients (n = 109), C In the subgroup American Joint Committee of Cancer (AJCC)8th edition stage I+II (n = 369); D In the subgroup AJCC8th I+II (n = 43). [file 12885_2023_11526_MOESM3_ESM.pptx]

## Slide 1
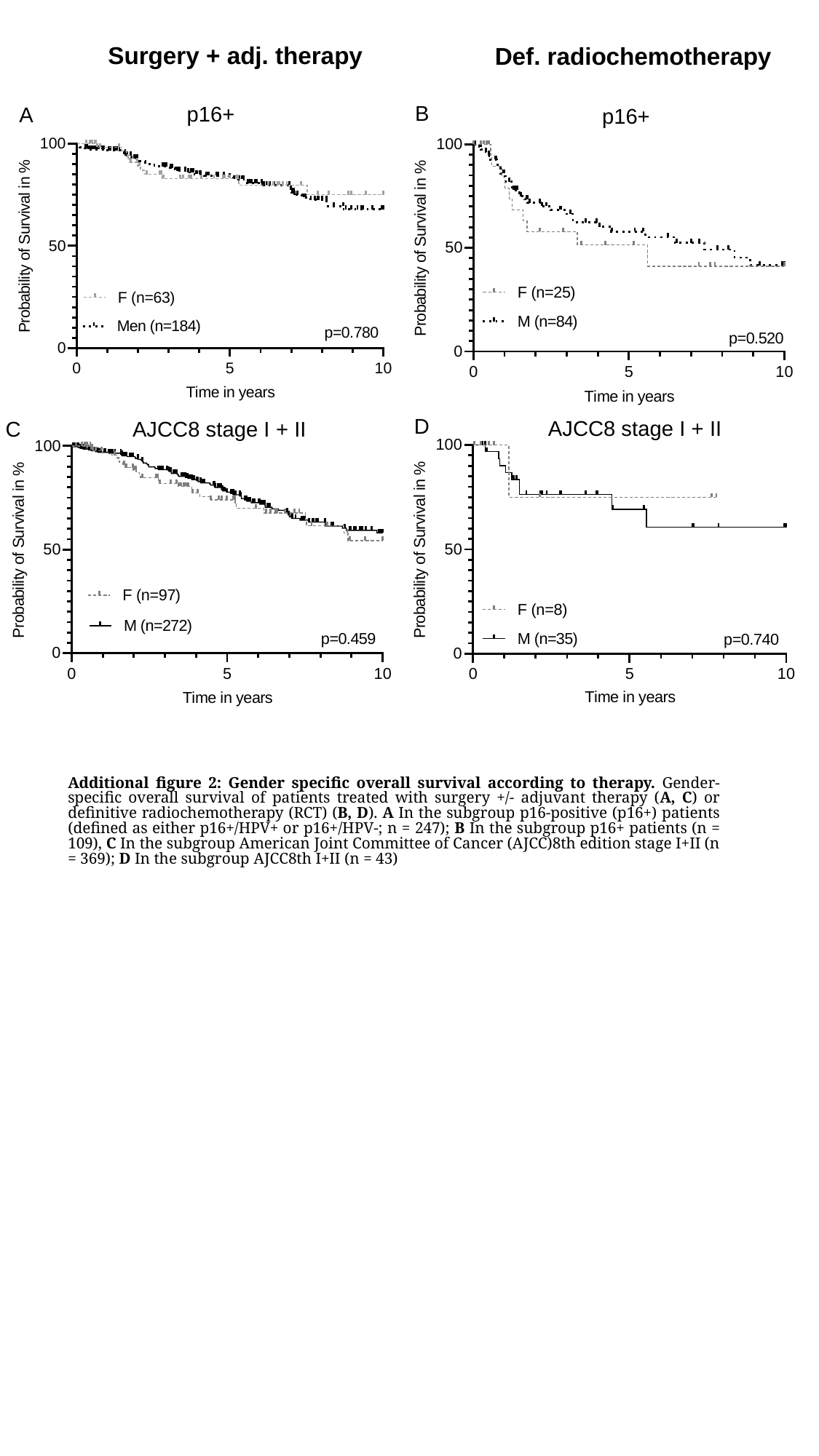

Surgery + adj. therapy
Def. radiochemotherapy
B
A
D
C
p16+
p16+
AJCC8 stage I + II
AJCC8 stage I + II
Additional figure 2: Gender specific overall survival according to therapy. Gender-specific overall survival of patients treated with surgery +/- adjuvant therapy (A, C) or definitive radiochemotherapy (RCT) (B, D). A In the subgroup p16-positive (p16+) patients (defined as either p16+/HPV+ or p16+/HPV-; n = 247); B In the subgroup p16+ patients (n = 109), C In the subgroup American Joint Committee of Cancer (AJCC)8th edition stage I+II (n = 369); D In the subgroup AJCC8th I+II (n = 43)
