# Supplementary material for: Sex-specific aspects in patients with oropharyngeal squamous cell carcinoma: a bicentric cohort study
Source: BMC Cancer. 2023 Nov 2;23:1054. doi: 10.1186/s12885-023-11526-6 (PMC10621233; doi:10.1186/s12885-023-11526-6)
Supplement: Supplementary file 4 — Additional file 4. Overall Survival in Human papillomavirus positive vs. Human papillomavirus negative cohort. A In the male cohort (n = 957); B In the female cohort (n = 281); C + D In the total cohort (n=1238). HPV-negative defined as either p16-/HPV-, p16-/HPV+ and p16+/HPV-; HPV-positive defined as p16+/HPV+; ** = p. [file 12885_2023_11526_MOESM4_ESM.pptx]

## Slide 1
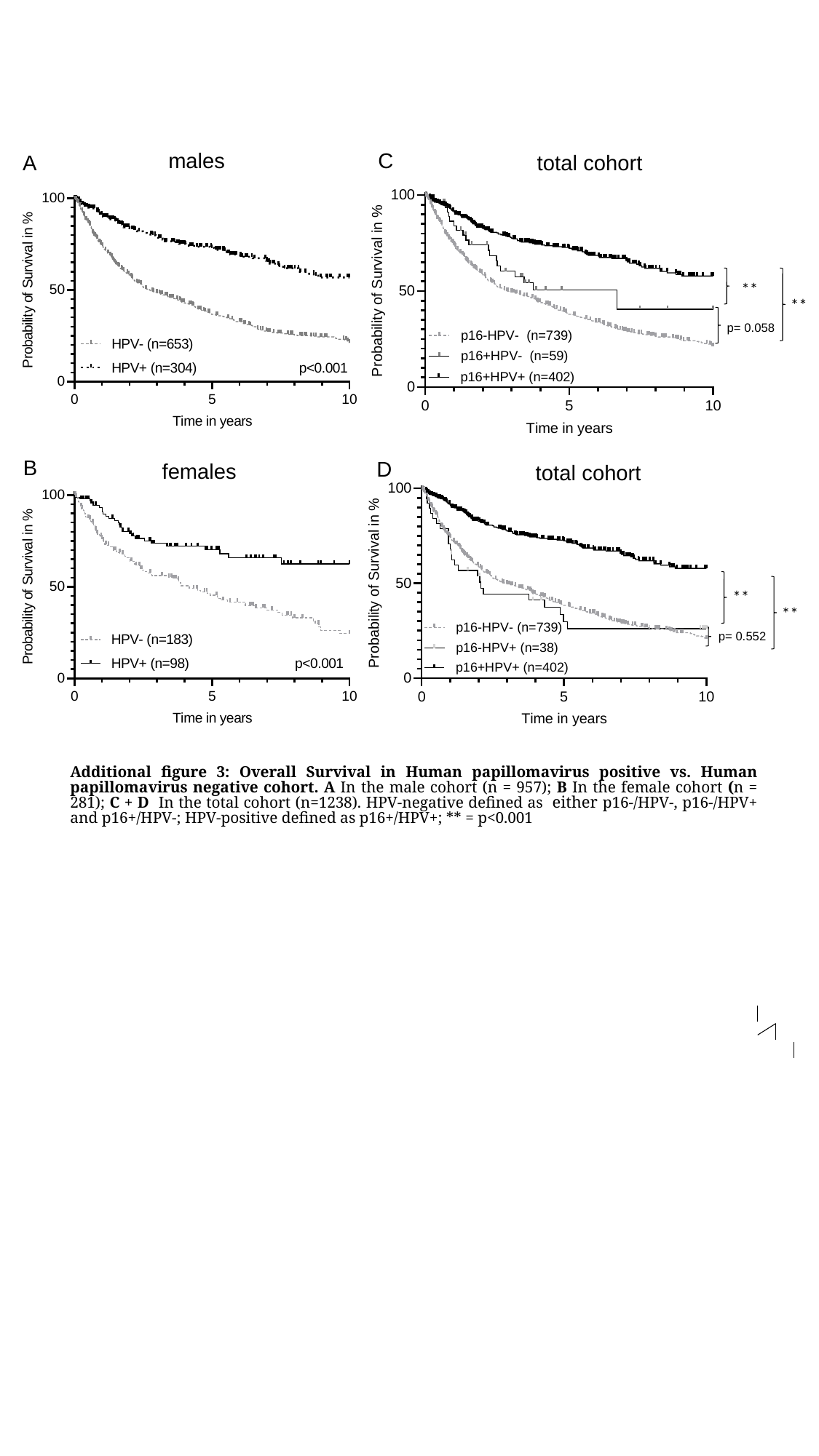

males
C
A
females
total cohort
B
**
**
p= 0.058
D
total cohort
**
**
p= 0.552
Additional figure 3: Overall Survival in Human papillomavirus positive vs. Human papillomavirus negative cohort. A In the male cohort (n = 957); B In the female cohort (n = 281); C + D In the total cohort (n=1238). HPV-negative defined as either p16-/HPV-, p16-/HPV+ and p16+/HPV-; HPV-positive defined as p16+/HPV+; ** = p<0.001
